# Genetic Variants of C-5312T *REN* Increased Renin Levels and Diastolic Blood Pressure Response to Angiotensin Receptor Blockers

**DOI:** 10.1155/2015/930631

**Published:** 2015-10-01

**Authors:** Mohammad Saifur Rohman, Ika Arum Dewi Satiti, Nashi Widodo, Mifetika Lukitasari, Hidayat Sujuti

**Affiliations:** ^1^Department of Cardiology and Vascular Medicine, Faculty of Medicine, Saiful Anwar General Hospital, Brawijaya University, Malang 65141, Indonesia; ^2^Biomedical Sciences, Faculty of Medicine, University of Brawijaya, Malang 65141, Indonesia; ^3^Biology Department, Faculty of Mathematics and Sciences, University of Brawijaya, Malang 65141, Indonesia; ^4^Nursing Department, Faculty of Medicine, University of Brawijaya, Malang 65141, Indonesia; ^5^Biochemistry Laboratory, Faculty of Medicine, University of Brawijaya, Malang 65141, Indonesia

## Abstract

Renin catalyzes the cleavage of angiotensinogen into angiotensin I. Genetic variant C-5312T of renin enhancer has been reported to increase in vitro renin gene transcription. However, no obvious in vivo study was performed to see the renin level in C-5312T when treated with angiotensin receptor blockers (ARB). Therefore, this study aimed to investigate the serum renin level and blood pressure response in ARB treated hypertensive patients. Single nucleotide polymorphism (SNP) of C-5312T was identified in 55 hypertensive patients by using multiplex PCR and renin serum level was assayed by ELISA. The data showed that the increase of serum renin levels after 5 months of ARB treatment was significantly higher in patients with CT/TT genotype (10 pg/mL) than those with CC genotype (4.08 pg/mL) (*P* = 0.025). Hypertensive patients with CT/TT genotypes also showed less diastolic pressure reduction than CC genotypes in hypertensive patients with valsartan treatment (*P* = 0.04) or telmisartan treatment (*P* = 0.03). Finally, these findings suggested that SNP of C-5312T *REN* enhancer might contribute to higher increased renin serum levels and less diastolic blood pressure response to ARB treatment.

## 1. Introduction

The renin-angiotensin system (RAS) plays important roles in the regulation of electrolytes, blood pressure (BP), and atherosclerosis [1]. Renin catalyzes the first and rate limiting step of the cascade, the conversion of angiotensinogen to angiotensin I [2]. Genetic variants of this system have been developed to test their association with cardiovascular and renal condition [3]. It has been reported that genetic variant* REN* of C-5312T might be functional and lead to changing renin transcriptions in hypertensive patients. Increased renin gene transcription may increase the concentration of renin in the circulation thereby increasing the activation of the renin-angiotensin system [4, 5].

Angiotensin receptor blockers are the treatment of hypertension based RAS blockade where a number of large-scale prospective studies have proven that this medication have favorable effects of cardiovascular and renal conditions. Our previous study in Saiful Anwar outpatient clinic revealed that 42% of hypertensive patients were treated by ARB whereas only 21.8% of the patients achieved the blood pressure target [6]. These facts rise an intriguing question whether the variety of ARB therapy response exists among hypertensive patients.

Previous study has suggested that genetic variant* REN* C-5312T significantly resulted in an increase of plasma renin activity (PRA) on 3 months' ARB administration Japanese hypertensive patients [7]. However, the presence of renin genetic variants and their effect on Indonesian hypertensive patients and serum renin levels response to ARB remain unclear. Hence, this study was conducted to investigate the presence of renin genetic variants on enhancer region of rennin gene and their effect on renin serum level after ARB administration.

## 2. Material and Methods

This study was conducted in RSUD Dr. Saiful Anwar Malang during July–December 2014. Adult hypertensive patients with creatinine < 2.5 mg/dL and good medication adherence were recruited. Antihypertensive medication adherence was measured by Morisky Medication Adherence Score (MMAS) [8–10]. Compliance and blood pressure were observed during 5 months of follow-up. Then salt intake was assessed by Food Frequency Questioner (FFQ) [11].

Hypertensive patients with massive bleeding, hepatic failure, renal failure, pregnancy, and known estrogen/corticosteroid therapy were excluded from this study. Blood samples were collected at the baseline and after receiving valsartan (25 patients) and telmisartan (30 patients) at least for 5 months. Renin serum was assayed by indirect ELISA method.

### 2.1. Genetic Variant Analysis

Genomic DNA samples were isolated using a DNA extraction kit (Geneaid). Renin gene amplification was assayed by multiplex PCR with the followingprimers sense oligo 5′-CGT AGT GCC ATT TTT AGG AAC′-3′ and antisense oligo 5′-AAC ACC AAA GCA GGC-3′ [4] with additional primers originally designed by us from the adjacent sequence [12], which were the following: sense oligo 5′-GCA GTC TCT GTA AGT GCC AC-3′ and antisense oligo 5′-CCA AAG CAG GCT TAA TCT CA-3′. The program consisted of 35 cycles of denaturation at 95°C temperature for 30 seconds, annealing at a temperature gradient 48–58°C for 30 seconds and extension at 72°C for 1 min followed by a final extension at 72°C for 10 minutes.

### 2.2. Statistical Analysis

Phenotypic data are expressed as mean ± SD, as median [interquartile range], or as numbers (percentages). The differences between two paired continuous variables in genotype CC and genotype CT/TT were analyzed by paired *t*-test. The differences of renin levels between genotypes CC and CT/TT renin gene were analyzed by unpaired *t*-test. And the differences of characteristic baseline between two genotypes groups were assased by chi-square test as appropriate with considered significant value *P* < 0.05.

## 3. Results

### 3.1. Genetic Variants of* REN* C-5312T and Baseline Characteristic

A total of 55 subjects received monotherapy with valsartan or telmisartan for 5 months and completed the study. The baseline demographic, clinical, and biochemical characteristics of the subjects were almost balanced among genotypes of* REN* C-5312T. The distributions of genotypes for these SNPs were all in Hardy–Weinberg Equilibrium. From the above baseline characteristics significant differences of age, history of hypertension, BMI, dietary salt, urea, and creatinine levels between genotype CC and CT/TT were not found (Table 1). However, females more frequently have TT/CT genotype (*P* = 0.008) (Table 1).

### 3.2. Genotype Frequency of Renin C-53212T

**Figure 1:**
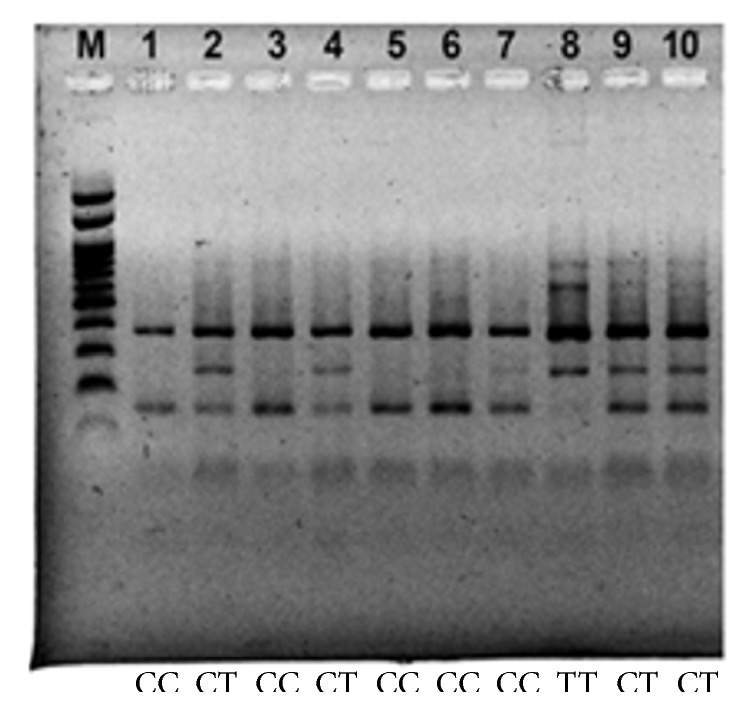
Renin gene C-5312T amplification. Photos of electrophoresis results (PCR product of 10 hypertensive patients), with an ethidium bromide dye on agarose 1.5%. From the left, image (M) is marker, and wells 1 to 10 are a band of renin gene polymorphism with genotypes CC, CT, and TT.

DNA amplification product of the renin gene C-5312T carried by PCR multiplex was then tested qualitatively using 1.5% agarose (Figure 1). The results of the multiplex PCR analysis on 55 samples showed that the respondents with CT/TT genotypes (64.3%) are larger than respondents with CC genotypes (35.7%) and further analysis used chi-square test with significant difference found between two genotypes frequencies (*P* = 003).

### 3.3. Association of Renin Levels of C-5312T and ARB Therapy in Hypertensive Patients

Renin levels results were obtained from measurements of serum hypertensive patients with indirect Elisa method. The result showed that there was a difference in the changes of renin levels in C-5312T genotypes in response to telmisartan and valsartan therapy. CT/TT genotype has a higher increase (10 pg/mL) of renin levels than CC genotype (4.06 pg/mL) after 5 months of telmisartan therapy, but not valsartan, as shown in Tables 2 and 3.

### 3.4. Genetic Variants of C-5312T* REN* and Reduction in Blood Pressure with ARB

Changes in blood pressure genetic variation of C-5312T* REN* are summarized in Tables 4 and 5. Among the 55 hypertensive patients, changes in diastolic blood pressure (DBP) were significantly different according to the genetic variant of renin C-5312T in response to telmisartan and valsartan therapy. This result showed that the reductions in DBP in CC genotype were significantly larger (10 mmHg) than CT/TT genotype (15.3 mmHg) in hypertensive patients after 5 months of valsartan therapy. CC genotype also showed a larger reduction of DBP (8 mmHg) than CC/TT genotype (12 mmHg) in hypertensive patients after 5 months of valsartan therapy.

### 3.5. Association of Renin Levels of C-5312T and Salt Intake in Hypertensive Patients

It is known that salt is one of the important elements in the regulation of renin secretion, so in this study, we analyzed the differences of renin levels in hypertensive patients with nonsalt diet and dietary salt. This study shows that renin levels in patients with salt diet were higher than patients with nonsalt diet (*P* = 0.021) (Tables 6 and 7). This result proves that salt is a regulator of renin secretion [11, 13].

Then to determine the difference of renin levels between CT/TT genotypes and CC genotypes based on salt intake and its responses to early ARB therapy, we analyzed the differences of renin levels between genotype CC and CT/TT in patients with salt dietary. From the analysis we obtained that renin levels of genotype CT/TT were significantly higher than CC genotype in patients with salt dietary. It is suggested that CC genotype is a predictor responder ARB in early therapy.

## 4. Discussion

In the RAS system, renin is the main enzyme activation system that started this. Renin gene has a major role in controlling blood pressure. Renin catalyzes the first and rate limiting step of the cascade, the conversion of angiotensinogen to angiotensin I [2, 14, 15]. Renin gene transcription level is influenced by many factors such as the presence of distal enhancers renin gene and the presence of polymorphisms in the area. Several studies have identified SNPs in genes related to the renin hypertension [15–17]. Previous research reported that the renin gene polymorphism at position C-5312T area renin gene enhancer affects the renin gene expression. Fuchs et al. revealed that variant genetic renin gene -5312T increases renin gene transcription level of 45% in comparison with genotype -5312C.

There are three transcription factors that are identified in the renin gene enhancers including NRF-2, Sp1, and AP-1. All three proteins may be regarded as a functional transcription factor in the renin gene enhancer. This is evidenced from previous research by Germain et al. (1998) which revealed that the binding of AP-1 by binding its site can initiate transcription of the renin gene enhancer [17]. Similarly, the presence of mutations or deletions in the site NRF-2 can reduce the activity of the renin gene transcription enhancers as much as 89%, whereas mutations in the Sp1 sites can reduce the activity of the renin gene transcription by 40% [18, 19]. Transcription factor SP-1 also has the same consensus with genetic variants renin -5312C and -5312T. Activation of this transcription factor is influenced by several factors including the binding of angiotensin II (Ang-II) with AT1R which can be activated by several signaling and MAPK pathways. This pathway can induce phosphorylate coactivator of Nrf2, AP-1, and SP-1 transcription factor to initiate* REN* gene basal transcriptions [20].

The therapeutic effect may be supported by the presence of AT1R signaling blockade, where this signaling can inhibit the MAPK pathway, and activation of transcription factor (SP-1, AP-1, and Nrf2). In this study a significant difference in the change in renin levels was found in early therapy and after therapy between CC genotype and genotype CT/TT. CT/TT genotype has a higher increase of renin levels than CC genotype. This may be caused by the change from C to T base renin gene enhancer-5312 position, which can increase transcription factor binding to its binding site. A previous study analyzed the pattern of binding of transcription factor SP-1 to site* REN* gene enhancer-5312, proving that the binding energy is greater on the T allele than the C allele.

Previous study also showed that genetic variant of renin contributes to BP variation in a Caucasian population. Carriers of the renin -5312T allele had higher systolic and diastolic BP in hypertensive patients on 3 months of ARB therapy. This study suggested that CC genotype is a predictor responder ARB therapy. However, it is widely known that ARB causes a negative feedback mechanism in renin release. Angiotensin receptor blocker (ARB) acts as an antagonist AT1 receptor that prevents binding of angiotensin II to AT1 receptor, which acts as inhibition of renin production by juxtaglomerular cells in the kidney. The admission of ARB causes the loss of stimulation of AT1 receptors on juxtaglomerular cells that initiate negative feedback to improve the activity of the renin which can increase plasma renin activity and also increase formation of angiotensin I.

## 5. Conclusion

In this study it was indicated that CT/TT genotype has a significantly higher renin levels than CC genotype (*P* = 0.025). Then, the reductions of DBP in C allele homozygotes were significantly larger than those in T allele carriers in hypertensive patients after 5 months of valsartan or telmisartan treatment. These findings suggested that SNP of C-5312T* REN* enhancer might contribute to higher increased renin serum levels and less diastolic blood pressure response to ARB treatment.

## Limitations

Several limitations of this study should be noted. The sample size we have used is still relatively small to represent generalization of Indonesian population. Other genetic and nongenetic factors that can influence renin levels are not evaluated in this study.

## Figures and Tables

**Table 1:** Baseline characteristic of hypertensive patients.

Variable	TT/CT	CC	*P*
Age, years	58.6 ± 9.17	54.5 ± 7.64	0.062
Sex, *n* (%)			
Male	15 (41.6)	15 (78.9)	0.008^*∗*^
Female	21 (78.9)	4 (21.1)	
Duration of suffering hypertension, months	84.86 ± 42.06	64.80 ± 78.85	0.578
BMI	27 (3.46)	26,2 (4.19)	0.496
Salt diet			
Yes	13 (68.4)	20 (55.5)	0.138
No	7 (36.8)	16 (44.4)	
Creatinine, mg/dL	1.08 ± 0.27	1.01 ± 0.28	0.40
Urea, mg/dL	26 ± 4.75	30.3 ± 15.1	0.22

Data are means ± SD or medians (interquartile ranges). ^*∗*^The differences between baseline characteristic variables for genotypes CC and CT/TT were analyzed by chi-square test as appropriate.

**Table 2:** Differences of renin levels (telmisartan therapy).

Variable	Genotypes		*P* value
	CC	CT/TT	
Early therapy	37.32 ± 9.04	37.64 ± 9.50	0.944
After therapy	41.80 ± 10.09	47.64 ± 10.02	0.025^*∗*^
Δ delta	4.08 ± 1.35	10 ± 2.34	0.025^*∗*^

Data are means ± SD or medians (interquartile ranges). ^*∗*^The differences between renin levels variable for genotypes CC and CT/TT were analyzed by independent *t*-test as appropriate.

**Table 3:** Differences of renin levels (valsartan therapy).

Variable	Genotypes		*P* value
	CC	CT/TT	
Early therapy	37.03 ± 6.96	38.76 ± 5.59	0.44
After therapy	43.67 ± 10.49	47.81 ± 9.02	0.32
Δ delta	6.64 ± 1.25	9.05 ± 3.44	0.40

Data are means ± SD or medians (interquartile ranges). The differences between renin levels variable for genotypes CC and CT/TT were analyzed by independent *t*-test as appropriate.

**Table 4:** Genetic variants of the *REN* and reduction in blood pressure (valsartan therapy).

*REN* C-5312T	SBP (mmHg)		*P*	DBP (mmHg)		*P* ^**∗**^
	Baseline	ARB		Baseline	ARB	
CT/TT	155.2 ± 15.3	135 ± 13.6	0.56	93.20 ± 15.5	83.2 ± 13.6	0.04
CC	140.65 ± 19.03	120.5 ± 20.5		92.45 ± 15.23	80.1 ± 14.6	

Data are means ± SD. Statistical analysis of the difference was performed by independent *t*-test as appropriate. ^*∗*^ *P* value was calculated for the statistics showing the interactive effect of ARB administration and category in patients on DBP.

**Table 5:** Genetic variants of the *REN* and reduction in blood pressure (telmisartan therapy).

*REN* C-5312T	SBP (mmHg)		*P*	DBP (mmHg)		*P* ^*∗*^
	Baseline	ARB		Baseline	ARB	
CT/TT	150.5 ± 18.4	130.5 ± 13.6	0.66	90.20 ± 15.5	82.2 ± 15.4	0.03
CC	145.7 ± 17.9	125 ± 20.5		92.45 ± 15.23	80.1 ± 15.6	

Data are means ± SD. Statistical analysis of the difference was performed by independent *t*-test as appropriate. ^*∗*^ *P* value was calculated for the statistics showing the interactive effect of ARB administration and category in patients on DBP.

**Table 6:** Difference of renin levels based on salt intake.

Salt diet	Baseline renin levels	*P* value
No (19)	33.5 ± 7.84	0.021
Yes (36)	40.4 ± 8.64	

Data are means ± SD or medians (interquartile ranges). ^*∗*^The differences between renin levels variable for salt and nonsalt diet hypertensive patients were analyzed by independent *t*-test as appropriate.

**Table 7:** Difference of renin levels based on genotype in salt diet patients.

Genotype	Baseline levels renin	*P* value
CT/TT (13)	39.5 ± 8.84	0.027
CC (7)	33.4 ± 7.34	

Data are means ± SD or medians (interquartile ranges). ^*∗*^The differences between renin levels variable for genotypes CC and CT/TT were analyzed by independent *t*-test as appropriate.

## References

[1] Huang C.-C., Leu H.-B., Huang P.-H., Wu T.-C., Lin S.-J., Chen J.-W. (2013). Baseline serum aldosterone-to-renin ratio is associated with the add-on effect of thiazide diuretics in non-diabetic essential hypertensives. *Acta Cardiologica Sinica*.

[2] van Vark L. C., Bertrand M., Akkerhuis K. M. (2012). Angiotensin-converting enzyme inhibitors reduce mortality in hypertension: a meta-analysis of randomized clinical trials of renin-angiotensin-aldosterone system inhibitors involving 158 998 patients. *European Heart Journal*.

[3] Lukitasari M., Rohman M. S., Hendrawan D. Achievement of blood pressure target with angiotensin blockage based therapy in outpatient clinic.

[4] Konoshita T., Kato N., Fuchs Ś. (2009). Genetic variant of the renin-angiotensin system and diabetes influences blood pressure response to angiotensin receptor blockers. *Diabetes Care*.

[5] Chang S.-N., Lin J.-W., Juang J.-M., Tsai C.-T., Hwang J.-J., Chiang F.-T. (2010). Association between genetic polymorphisms in the renin-angiotensin system and systolic heart failure revised by a propensity score-based analysis. *Cardiology*.

[6] Lloyd-Jones D. M., Evans J. C., Levy D. (2005). Hypertension in adults across the age spectrum: current outcomes and control in the community. *Journal of the American Medical Association*.

[7] Lu F.-H., Tang S.-J., Wu J.-S., Yang Y.-C., Chang C.-J. (2000). Hypertension in elderly persons: its prevalence and associated cardiovascular risk factors in Tainan City, Southern Taiwan. *Journals of Gerontology A*.

[8] Filigheddu F., Argiolas G., Bulla E. (2008). Clinical variables, not RAAS polymorphisms, predict blood pressure response to ACE inhibitors in Sardinians. *Pharmacogenomics*.

[9] Fan X., Wang Y., Sun K. (2007). Polymorphisms of ACE2 gene are associated with essential hypertension and antihypertensive effects of Captopril in women. *Clinical Pharmacology & Therapeutics*.

[10] Sun B., Pojoga L., Chamarthi B. (2011). Renin gene polymorphism: its relationship to hypertension, renin levels and vascular responses. *Journal of the Renin-Angiotensin-Aldosterone System*.

[11] Forster H., Fallaize R., Gallagher C. (2014). Dietary intake estimation: food me food frequency quetsioner. *Journal of Medical Internet Research*.

[12] Fuchs S., Philippe J., Germain S. (2002). Functionality of two new polymorphisms in the human renin gene enhancer region. *Journal of Hypertension*.

[13] Cagnoni F., Njwe C. A. N., Zaninelli A. (2010). Blocking the RAAS at different levels: an update on the use of the direct renin inhibitors alone and in combination. *Vascular Health and Risk Management*.

[14] Verdecchia P., Angeli F., Mazzotta G., Gentile G., Reboldi G. (2008). The renin angiotensin system in the development of cardiovascular disease: role of aliskiren in risk reduction. *Vascular Health and Risk Management*.

[15] Huang C., Bang H., Leu T., Huang P., Cheng W. T., Jong L. S. (2013). Baseline serum aldosterone-to-renin ratio is associated with the add-on effect of thiazide diuretics in non-diabetic essential hypertensives. *Acta Cardiologica Sinica*.

[16] Harrison-Bernard L. M., Navar L. G., Ho M. M., Vinson G. P., El-Dahr S. S. (1997). Immunohistochemical localization of ANG II AT1 receptor in adult rat kidney using a monoclonal antibody. *The American Journal of Physiology—Renal Physiology*.

[17] Germain S., Bonnet F., Philippe J., Fuchs S., Corvol P., Pinet F. (1998). A novel distal enhancer confers chorionic expression on the human renin gene. *The Journal of Biological Chemistry*.

[18] Friis U. G., Madsen K., Svenningsen P. (2009). Hypotonicity-induced renin exocytosis from juxtaglomerular cells requires aquaporin-1 and cyclooxygenase-2. *Journal of the American Society of Nephrology*.

[19] Poch E., González D., Giner V., Bragulat E., Coca A., de la Sierra A. (2001). Molecular basis of salt sensitivity in human hypertension: evaluation of renin-angiotensin-aldosterone system gene polymorphisms. *Hypertension*.

[20] Ekker M., Tronik D., Rougeon F. (1989). Extra-renal transcription of the renin genes in multiple tissues of mice and rats. *Proceedings of the National Academy of Sciences of the United States of America*.

